# Inhibition of Horse Liver Alcohol Dehydrogenase by Methyltin Compounds

**DOI:** 10.1155/BCA.2005.191

**Published:** 2005

**Authors:** Pavel V. Bychkov, Tatyana N. Shekhovtsova, Elena R. Milaeva

**Affiliations:** M.V. Lomonosov Moscow State University Chemistry Department Leninskie Gory Moscow 119992 Russia

**Keywords:** alcohol dehydrogenase, methyltin compounds, NAD, inhibition type

## Abstract

The study of inorganic tin (SnCl_2_, SnCl_4_) and methyltin compounds (MeSnCl_3_, Me_2_SnCI_2_, Me_3_SnCl) effects on the enzymatic activity of alcohol dehydrogenase (ADH) in the reaction of ethanol oxidation has been carried out. The experimental results of the study show that inorganic tin and methyltin substances induce slight inhibition of the catalytic activity of horse liver alcohol dehydrogenase (HLADH), unable to be improved during pre-incubation with the enzyme. The conditions for carrying out the kinetic investigation of the mentioned phenomenon were optimized and as it turned out the mechanism of methyltin trichloride action, as the most effective methyltin inhibitor, is more complex than the proposed interaction of the metal atom with SH-groups of the enzyme protein. It was demonstrated that the tin compounds act in the same manner as methylmercury compounds and might serve as oxidative agents towards the co-enzyme NADH. Kinetic data on MeSnCl_3_ were calculated. Data acquired on NAD-dependent ADH from horse liver and those regarding NAD-dependent LDH from sturgeon liver were compared.


### Inhibition of Horse Liver Alcohol Dehydrogenase by Methyltin Compounds

Pavel V. Bychkov, Tatyana N. Shekhovtsova*, Elena R. Milaeva

*M.V. Lomonosov Moscow State University, Chemistry Department,*
*Leninskie Gory, 119992 Moscow, Russia. Fax: +7 095 939 4675;*
*e-mail: shekhov@analyt.chem.msu.ru*

### ABSTRACT

The study of inorganic tin ($SnCl_2$, $SnCl_4$) and methyltin compounds ($MeSnCl_3$, $Me_2SnCl_2$, $Me_3SnCl$) effects on the enzymatic activity of alcohol dehydrogenase (ADH) in the reaction of ethanol oxidation has been carried out. The experimental results of the study show that inorganic tin and methyltin substances induce slight inhibition of the catalytic activity of horse liver alcohol dehydrogenase (HLADH), unable to be improved during pre-incubation with the enzyme. The conditions for carrying out the kinetic investigation of the mentioned phenomenon were optimized and as it turned out the mechanism of methyltin trichloride action, as the most effective methyltin inhibitor, is more complex than the proposed interaction of the metal atom with SH-groups of the enzyme protein. It was demonstrated that the tin compounds act in the same manner as methylmercury compounds and might serve as oxidative agents towards the co-enzyme NADH. Kinetic data on $MeSnCl_3$ were calculated. Data acquired on NAD-dependent ADH from horse liver and those regarding NAD-dependent LDH from sturgeon liver were compared.

**Keywords**: alcohol dehydrogenase, methyltin compounds, NAD, inhibition type.

### 1. INTRODUCTION

Due to the widespread industrial application of tin and its organic derivatives a considerable amount of these compounds has entered various ecosystems. Whereas inorganic tin compounds are considered to be non-toxic, the toxicological pattern of organotins is complex. They show specific toxic effects to different organisms even at very low concentrations /1/.

There are considerable variations in toxicity within the class of organotin compounds [$R_nSnX_{4-n}$ (n =.1 - 3)], depending both on the nature and number of organic groups R and on the nature of species formed in different media /2/. Special attention is given to methyl derivatives of tin [$(CH_3)_nSnX_{4-n}$ (n = 1 - 3)] since these organotins are formed in the environment as a result of biomethylation processes /3/.

The toxicity of organotins is associated with their ability to react with free SH-groups in proteins that leads to the inhibition of some enzymes activities, resulting in tissue-damaging effects. However there are data indicating the participation of organotin compounds in biochemical radical and redox processes which might involve the electron transfer from low molecular substances to organic derivatives of Sn(IV) as oxidants /4/. Co-enzyme NADH belongs to those substances which are oxidised by organotin and organomercury compounds /5/.

NAD-dependent alcohol dehydrogenases (ADH) are the enzymes catalyzing the first stage of ethanol oxidation as well as the oxidation of some other alcohols /6/. It was shown that the catalytic activity of ADH is inhibited sufficiently in the presence of methylmercury, as well as inorganic mercury /7/. On the other hand, the inhibitory effect of methyltin trichloride and trimethyltin chloride on NAD-dependent lactate dehydrogenase (LDH) was observed /8/. Moreover, methyltin trichloride turned out to be a purely non-competitive inhibitor of LDH.

The goal of this work was to study the influence of a series of methyltin compounds on the catalytic activity of alcohol dehydrogenase from horse liver (HLADH), to determine the inhibition type and clarify the mechanism of methyltin compounds inhibitory activity.

### 2. EXPERIMENTAL

### Materials and Instruments:

The following materials were obtained commercially and used as received: alcohol dehydrogenase from horse liver (Fluka AG), NAD (Sigma), $SnCl_2$, $SnCl_4$, $MeSnCl_3$, $Me_2SnCl_2$, $Me_3SnCl$ (Strem). Demineralized water purified with Simplicity Proto system (Millipore) was used. Aqueous solutions of HLADH were prepared by dissolving the precise quantity of the enzyme (E.C.1.1.1.1.) in phosphate buffer (pH 7.6). Aqueous NAD solutions were prepared by dissolution of the reagent in purified water. Ethanol solutions were prepared using 96% ethanol and purified water. The solutions of $SnCl_2$, $SnCl_4$, $MeSnCl_3$, $Me_2SnCl_2$, $Me_3SnCl$ were prepared directly before the analysis by dissolving in purified water. Spectrophotometric kinetic study was performed using Shimadzu UV-2201 spectrophotometer (Japan). The pH of aqueous solutions was measured with an accuracy of $\pm$ 0.005 using a potentiometer in the pH-meter mode Econics-Expert-001 (Russia).

### Reaction Conditions:

The following reagents addition sequence was used in all experiments without an inhibitor: the reaction medium (TRIS-HCl buffer solution), coenzyme ($NAD^+$), substrate (ethanol), enzyme (HLADH). Within the inhibition experiments, the sequence was the same with the exception of the inhibitor addition to the mixture of the coenzyme and substrate, followed up by HLADH addition. When using pre-incubation, the enzyme was mixed firstly with an inhibitor. After required time expired, the mixture was added to the reaction media (TRIS-HCl buffer solution) containing $NAD^+$ and ethanol.

### Spectrophotometric Measurements and Kinetic Study:

The rate of the indicator reaction was monitored spectrophotometrically in quartz cells at room temperature as the increase of optical density of the reaction solution (at 340 nm) due to the formation of NADH. The value of the indicator reaction initial rate ($V_0$, μM $min^{-1}$) was calculated according to the formula /9/:

$$V_0 = \Delta c/\Delta \tau = \Delta A \bullet l/\Delta t \bullet l \bullet \varepsilon = tg\alpha/\varepsilon \bullet l,$$

where *c* is the product concentration, *τ* – the reaction time, *ε* – NADH molar absorbance coefficient, *l* – cuvette length, *tgα* – a slope of the kinetic curve plotted as absorbance *vs.* time.

The steady-state kinetics of the enzymatic reaction for the evaluation of the inhibition type was studied on the base of the dependence of the initial reaction rate on the initial substrate concentration (varied from 3 to 30 mM) in the absence and presence of $MeSnCl_3$ (content varied from 0.03 to 0.1 mg/ml). The experimental data were processed by graphical method in Lineweaver-Burk coordinates (double reciprocal method) /10, 11/.

### 3. RESULTS AND DISCUSSION

### Optimization of the reaction conditions in the study of methyltins influence on the enzymatic activity of alcohol dehydrogenase.

The reaction of ethanol oxidation was chosen as the indicator one. The dependences of the reaction rate in the presence and/or in the absence of inhibitors on the pH medium and the reagents concentrations were studied. The conditions were considered to be optimum if the inhibition effect was clear and convenient for detection whereas the reaction rate was high enough to monitor it. The optimized conditions are: TRIS-HCl-buffer pH 9.5; concentrations: HLADH – 4 μM, $NAD^+$ – 0.6 mM, ethanol – 0.03 M.

It is known that methyltin compounds in aqueous solutions at pH higher than 6-8 exist as hydroxy species $MeSn(OH)_3$, $Me_2Sn(OH)_2$, $Me_3Sn(OH)$ /12/. However the hydrolysis pathways of organotins as well as species formation are more complicated /13/. It was found for instance that five different species are formed (with metal to $OH^-$ ion ratios 1:1, 1:2, 1:3, 1:4 and 2:5) at low $MeSnCl_3$ concentration /14/. On the other hand the interaction of organotins with proteins is supposed to be strongly dependent upon the number and nature of the organic groups bound to Sn atom. It is proposed that the nature of the anionic group (Cl or OH) is of only secondary importance /13/. The most important fact is that the mode of organotins binding to proteins moieties is determined by the strength of Lewis acidicity which is influenced also by the organic group nature bound to Sn atom /15/. Therefore the study of the influence of methyltin compounds on the enzyme activity might be carried out using chloride compounds comparing the role of methyl groups. At the same time one should keep in mind the complexity of the hydrolysis products formed in the medium. Thus it was important to determine the methyltins influence on the alcohol dehydrogenase activity depending on the number of methyl groups in the organotin molecules.

**Study of the influence of methyltin compounds on the HLADH catalytic activity.**

Methyl derivatives of Sn(IV) possessing various numbers of organic groups ($MeSnCl_3$, $Me_2SnCl_2$, $Me_3SnCl$) and inorganic compounds of Sn(II) and Sn(IV) ($SnCl_2$, $SnCl_4$) were selected for the investigation. The influence of all the substances mentioned above upon the enzyme activity was studied in a wide concentration range. It was shown that the catalytic activity of HLADH decreased significantly in the presence of high concentrations (about 1 mg/ml) of all methyltin compounds under investigation. At the same time it was found that 10 μg/ml content of $SnCl_2$ had no effect on HLADH activity, whereas $SnCl_4$ at the same concentration inhibited the enzyme sufficiently.

The dependence of the reaction rate on the enzyme-inhibitor pre-incubation time for all the substances under consideration was studied. The data obtained clearly showed that the inhibition effects were not affected by pre-incubation of the enzyme with the inhibitor, neither inorganic Sn(II)/Sn(IV), nor any methyltin compounds.

It is known that the toxicity of alkyl derivatives of tin decreases in the sequence $Me_3SnCl > Me_2SnCl_2 > MeSnCl_3$ with the latter believed to be a low-toxic compound /16/. The toxicity of tin compounds is usually attributed to the enzyme inhibition caused by the interaction of tin atom with SH-groups in the active site /1, 16/. On the other hand, methyltin compounds exhibiting clear-cut oxidative capacity which increases in the series $Me_3SnCl < Me_2SnCl_2 < MeSnCl_3$, can participate in biochemical redox reactions such as transformation of NADH to $NAD^+$ /5/.

The data on the inhibition of HLADH catalytic activity by methyltin compounds have shown that these substances exhibit loss of the enzymatic activity due to the interaction with thiol groups. It is also clear that the inhibition grows simultaneously with the oxidative capacity of the investigated substances. Taking into consideration the fact that equal amounts of Sn(II) and Sn(IV) showed drastically different inhibition levels of HLADH activity – less than 5% in the case of Sn(II) and 59% in the case of Sn(IV) – it should be stated that the oxidation state of the metal center also plays an important role in the inhibition of the enzyme activity.

The degree of HLADH inhibition in the presence of methyltins (I, %) was calculated according to the equation: I,% = (1–[$V_0$ in the presence of inhibitor]/[$V_0$ in the absence of inhibitor])•100%.

The values of the inhibition degree for a series of methyltin compounds follow the dependence on the number of methyl groups presented in the molecules and on the oxidative capacity for $MeSnCl_3$, $Me_2SnCl_2$ and $Me_3SnCl$ as 53, 40 and 20% respectively.

Rather poor inhibitory effects of all methyltin chlorides are assumed to refer to the simultaneous running of two competitive processes: interaction of methyltins with the enzymatic SH-groups in the protein and oxidation of NADH to $NAD^+$ by methyltins. The first process causes the inhibition of the enzyme (decreases the reaction rate due to the conformational changes in the enzyme structure), whilst the second one nominally speeds the reaction up by providing extra amounts of $NAD^+$. One can suppose that at the low concentrations of methyltin compounds the oxidation of NADH prevails; thus no inhibition can be detected. By increasing the methyltin content in the reaction mixture, the enzyme inhibition becomes more essential, hence can be seen clearly.

Thus it has been demonstrated that methyltin compounds cause the inhibition of HLADH catalytic activity due to coupling with SH-groups of the enzyme. It should be also noticed that $MeSnCl_3$ turned out to be the most effective methyltin inhibitor in accordance with the smallest steric hindrance. This idea has an approval in received data on inorganic tin chloride $SnCl_4$ inhibition being sufficiently greater than that of any methyltin compound used. It is worth mentioning that these data confute the proposition of $MeSnCl_3$ being one of the organotin species presenting the lowest toxicity in the series of methyltin chlorides.

### Estimation of the inhibition type of the HLADH catalytic activity by methyltin trichloride.

The type of HLADH inhibition by the methyltin compounds was studied by means of Lineweaver-Burk and Dixon /10, 11/ linearization methods (Figs. 1, 2) with methyltin trichloride as the model inhibitor and the most effective one studied in this work. It was shown that the studied methyltin compounds inhibited the enzyme incompetitively. This fact confirms the assumption that the mode of methyltin compounds action is more complex than the simple formation of the coordination bond between tin atom and sulphur atom in the protein moiety of the enzyme.

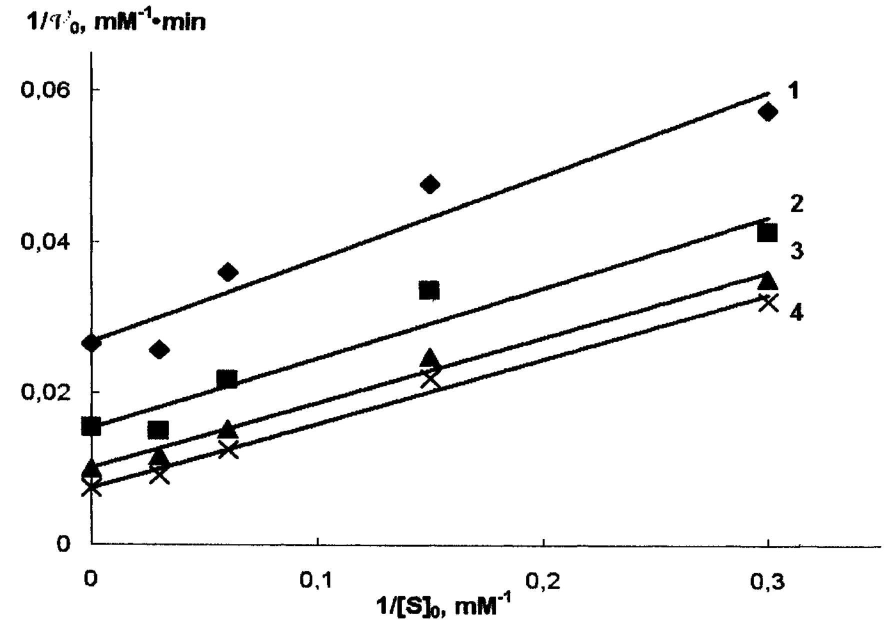

**Fig. 1:** The dependence of the reverse initial rate of the indicator reaction on the substrate (ethanol) concentration (Lineweaver-Burk coordinates) in the presence of methyltin trichloride (optimum conditions; $MeSnCl_3$ concentrations, mM: *1* – 0, *2* –2.1; *3* – 4.2; *4* – 6.3)

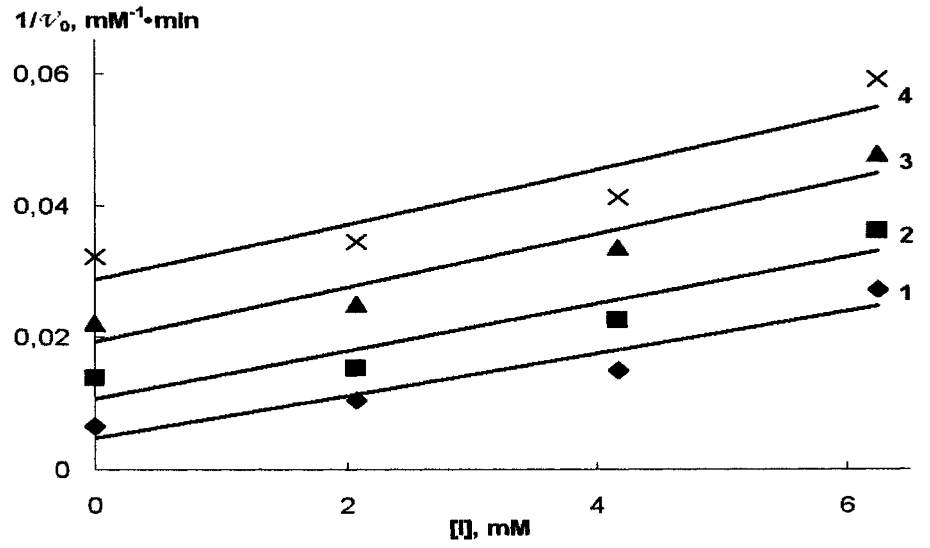

**Fig. 2:** The dependence of the reverse initial rate of the indicator reaction on $MeSnCl_3$ concentration with ethanol concentration varies (Dixon coordinates) (optimum conditions; ethanol concentrations, mM: *1* – 3.0; *2* – 6.5; *3* – 16.5; *4* – 33.0)

It should be mentioned that methyltin and methylmercury compounds inhibit HLADH catalytic activity in the same purely incompetitive manner /7/. The fact that methylmercury and Hg(II) species are much more effective HLADH inhibitors than methyltin and Sn(IV) species, could be accounted to the $pK_S$ values for the corresponding sulphides formed by these metal ions, that differ essentially (52 and 29 respectively /17/). The significant difference in the inhibition extent of methylmercury and methyltin compounds might be also seen by the comparison of their inhibition constants ($K_I$), being equal to 3.2 μM and 1.1 mM respectively /18/.

The results of this study show clearly that methyltin trichloride is an exceedingly poor inhibitor of HLADH in comparison with the other organometallic xenobiotics – methylmercury compounds. Inhibiting enzyme in the same manner, they have the extents of their action differing roughly 3000-fold. This might be explained by the significant increase of mercury atom affinity towards SH-groups in proteins when compared with tin atom.

### Comparison of the inhibitory effects of methyltin trichloride on the catalytic activity of two liver enzymes – ADH vs. LDH.

It was observed /5/ that methyltin trichloride did not hamper substrate binding with lactate dehydrogenase from liver of Russian sturgeon due to purely non-competitive type of inhibition. Indeed, as $MeSnCl_3$ concentration increases, maximum reaction rate ($v_{max}$) decreases whilst $K_M$ (Michaelis constant /11/) does not change and equals to 4.17 mM. In accordance with the data received in this work, $MeSnCl_3$, causing incompetitive inhibition of ADH, associates with the enzyme-substrate complex only but not with the enzyme itself. The last statement is elucidated also by the simultaneous decline of $v_{max}$ and $K_M$ values with the inhibitor concentration growth. Furthermore, the absence of the inhibition enhancement during ADH-$MeSnCl_3$ pre-incubation is another evidence of the question under consideration.

While comparing the conditions of the investigation of ADH and LDH inhibition (Table 1), one can suppose that $MeSnCl_3$ affects these cognate enzymes similarly. However, methyltin trichloride inhibits both mentioned enzymes in different ways (Table 2). The reason for this fact assumed to be found in their structure specificities. First of all there is a difference in their active sites configuration, and accessibility, and reactivity of SH-groups /14/. It should be also noted that additional vagueness is present because of using an *extract* of fish liver instead of isolated LDH /8/. Therefore it is difficult to determine whether $MeSnCl_3$ affected only LDH activity or was in conjunction with the extract media.

**Table 1**

Comparison of the investigation conditions of LDH [5] and ADH

| Enzyme | *LDH* | *ADH* |
|---|---|---|
| EC | **1.1.1.27** | **1.1.1.1** |
| Source | *Extract* from Russian sturgeon liver (*Asipenser gueldenstaedti* B.) | Horse liver, purchased from Fluka AG |
| Coenzyme, substrate | $NAD^+$, lactic acid | $NAD^+$, ethanol |
| Reaction conditions | pH 9.9-10.0<br>(0.10M glycine buffer)<br>$C_{NAD}$ = 0.5 mM<br>$C_S$ = 3 mM | pH 9.5<br>(0.05M TRIS-HCl buffer)<br>$C_{NAD}$ = 0.6 mM<br>$C_S$ = 30 mM |
| Reaction rate monitoring | Spectrophotometric<br>$\lambda_{max}$ = 340 nm | Spectrophotometric<br>$\lambda_{max}$ = 340 nm |
| Inhibition type | Purely noncompetitive | Purely incompetitive |

**Table 2**

Kinetic data on the inhibition of ADH and LDH [5] catalytic activity by $MeSnCl_3$ (optimum conditions of methyltin compound inhibition study)

| **Inhibitor concentration, mM** | | $K_I$, mM | | $K_M$, mM | |
|---|---|---|---|---|---|
| **ADH** | **LDH** | **ADH** | **LDH** | **ADH** | **LDH** |
| 0 | 0 | 1.13 | —* | 46.7±0.8 | 4.17 |
| 2.1 | 0.42 | | | 33.6±0.5 | |
| 4.2 | 0.75 | | | 21.1±0.3 | |
| 6.3 | 1.04 | | | 14.1±0.2 | |

* – was not determined

### 4. CONCLUSIONS

The experimental data of the study show that inorganic tin and methyltin substances induce slight inhibition of the catalytic activity of HLADH in the reaction of ethanol oxidation, unable to be improved during pre-incubation with the enzyme. The conditions for carrying out the kinetic investigation of the mentioned phenomenon were optimized and as it turned out the mechanism of methyltin trichloride action, as the most effective methyltin inhibitor, is more complex than the proposed interaction of the metal atom with SH-groups of the enzyme protein. It was demonstrated that the tin compounds act in the same manner as methylmercury compounds.

### ACKNOWLEDGEMENTS

This work was partially supported by the Russian Foundation for Basic Research (grants N 04-03-33116, N 03-03-32938).

### REFERENCES

1. T.R. Crompton. *Occurrence and Analysis of Organometallic Compounds in the Environment.* John Wiley, New York, 1998; p.47.
2. M. Hoch. *Appl. Geochem.*, **16**, 719 (2001).
3. P.J. Craig (Ed.), *The Biological Alkylation of Heavy Elements*, Royal Soc. Chem., London, 1988; p.343.
4. E.R. Milaeva, V.S. Petrosyan, N.T. Berberova, Yu.T. Pimenov and OL. Pellerito. *Bioinorg. Chem. Appl.* 2, (2004).
5. M.V. Medvedev, V. Yu. Tyurin., E.A. Rozhkova and E.R. Milaeva, *Khim. Getertsikl. Soedinen.*, 1999, 1036 [*Chem. Heterocycl. Compd.*, 1999 (*Internat. Ed.*.)].
6. P. Boyer, H. Lardy and K. Myrback, *The Enzymes.* New-York–London: Academic Press, 7, 25 (1963).
7. E.V. Zhmaeva, P.V. Bychkov and T.N. Shekhovtsova, Enzymatic determination of mercury(II) and methylmercury using alcohol dehydrogenases of different origin. / *Vestnik MGU. Khimiya..* **6,** 404 (2002).
8. M.N. Kolyada, Yu.T. Pimenov, N.T. Berberova, E.R. Milaeva, E.V. Kharitonashvili and V.S. Petrosyan, *Russian Chem. Bull. (Internat. Ed.),* **50**, 1485 (2001).
9. D. Perez-Bendito and M. Silva, *Kinetic Methods in Analytical Chemistry,* Ellis Horwood Limited Publishers. Chichester Halsted Press, 41 (1988).
10. T. Keleti, *Basic Enzyme Kinetics*, Academia Kiado, Budapest, 1990; p.234.
11. R.A. Copeland, *Enzymes: A Practical Introduction to Structure, Mechanism, and Data Analysis.* Willey-VCH. Inc. 2000; pp. 273-282.
12. R.S. Tobias. In *Organometals and Organometalloids: Occurrence and Fate in the Environment.* F.E.

Brinckman and J.M.Bellama (Eds.), *ACS Symp. Ser.* 82, 130, 1978.
13. L. Pellerito, L. Nagy. *Coord. Chem. Rev.* **224**, 111 (2002).
14. C. De Stefano, C. Foti, A. Gianguzza, F. Marrone and S. Sammartano, *Appl. Organomet. Chem.* **13**, 805 (1999).
15. R.S. Tobias, H.N. Farrer, M.B. Hughes, B.A. Nevett, *Inorg. Chem.* **5**, 2052 (1966).
16. P.J. Craig (Ed.), *Organometallic Compounds in the Environment*, Longman, Essex, 1986; p. 111.
17. Yu.Yu. Lur'e, *Spravochnik po Analiticheskoi Khimii* (*Handbook of Analytical Chemistry*), Moscow, Khimiya, 1989; p.95.
18. M.T. Musmeci, G. Madonia, M.T. Lo Giudice, A. Silvestri, G. Ruisi and R. Barbieri, *Appl. Organomet. Chem.*, **6**, 127 (1992).

